# Supplementary material for: Breaching the Barrier: Genome-Wide Investigation into the Role of a Primary Amine in Promoting E. coli Outer-Membrane Passage and Growth Inhibition by Ampicillin
Source: Microbiol Spectr. 2022 Nov 21;10(6):e03593-22. doi: 10.1128/spectrum.03593-22 (PMC9769794; doi:10.1128/spectrum.03593-22)
Supplement: Supplemental file 2 — Supplemental material. Download spectrum.03593-22-s0002.pdf, PDF file, 0.2 MB [file spectrum.03593-22-s0002.pdf]

**Supplemental material**

# Breaching the barrier: genome-wide investigation into the role of a primary amine in promoting *E. coli* outer-membrane passage and growth inhibition by ampicillin

Claire Maher[1,2], Ram Maharjan[2,3], Geraldine Sullivan[2,3], Amy K. Cain[2,3] and Karl A. Hassan[1,2*]

1. College of Engineering, Science and Environment, University of Newcastle, Australia

2. ARC Centre of Excellence in Synthetic Biology, Macquarie University, Australia

3. School of Natural Sciences, Macquarie University, Australia

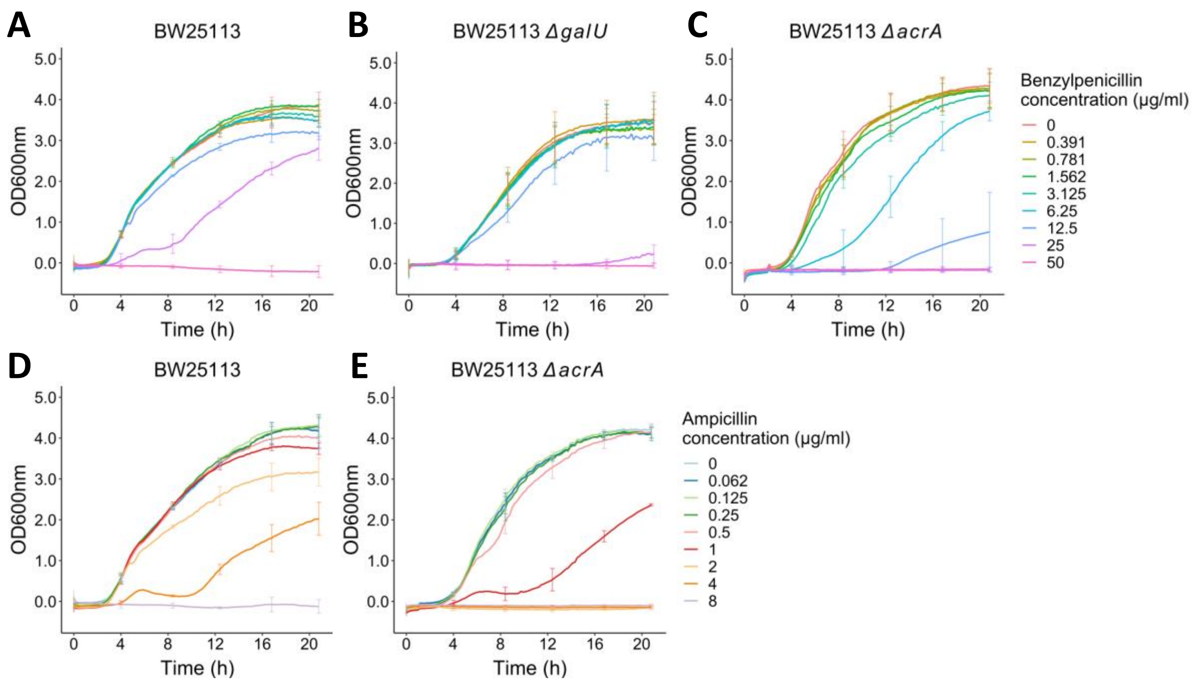


**Figure S1**. **Effects of gene deletions on BW25113 growth**. Deletion of *galU* (B) or *acrA* (C) inhibits BW25113 growth under benzylpenicillin treatment. Deletion of *acrA* also inhibits growth under ampicillin treatment (E). Deletion mutants were obtained from the Keio collection (Baba et al., 2006). Error bars represent SEM (n = 3).

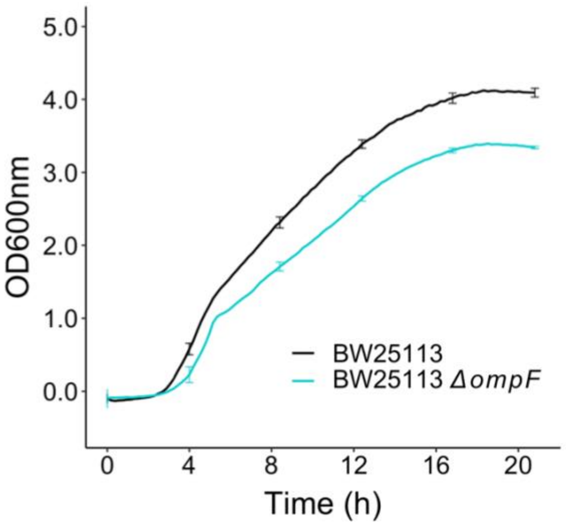


**Figure S2**. **Effect of *ompF* deletion on BW25113 growth**. Deletion of *ompF* inhibits growth under non-selective conditions. The *ompF* deletion mutant was obtained from the Keio collection (Baba et al., 2006). Error bars represent SEM (n = 3).

**Supplementary data file 1.** Fold changes in transposon mutant abundance for genes in the *E. coli* BW25113 genome between the antibiotic treated cell populations and untreated controls.
